# ABO Discrepancy in a Patient With Plasma Cell Myeloma

**DOI:** 10.7759/cureus.67096

**Published:** 2024-08-17

**Authors:** Sumaiyah Adzahar, Adibah Daud, Syamihah Mardhiah A Razak, Kamariah Abdul Jalil, Mohammad Hudzaifah Nordin, Muhammad 'Aqil Nazahah Mohamad Mustafa, Daniel Hazim Mohd Shukri, Azzahra Azhar, Sharifah Sakinah Syed Abdul Rahman, Razan Hayati Zulkeflee

**Affiliations:** 1 Department of Pathology and Medical Laboratory, Faculty of Medicine, Hospital Universiti Sultan Zainal Abidin, Kuala Terengganu, MYS; 2 Faculty of Medicine, Universiti Sultan Zainal Abidin, Kuala Terengganu, MYS; 3 Department of Pathology and Medical Laboratory, Hospital Universiti Sultan Zainal Abidin, Kuala Terengganu, MYS; 4 Department of Pathology and Medical Laboratory, Hospital Universiti Sultan Zainal Abidin, Terengganu, MYS; 5 Department of Hematology, School of Medical Sciences, Universiti Sains Malaysia, Kota Bharu, MYS

**Keywords:** malignancy, subgroup b, blood group, abo discrepancies, plasma cell myeloma

## Abstract

ABO discrepancies in plasma cell myeloma (PCM) present unique challenges in blood typing tests and transfusion management. We present the case of a 51-year-old male with PCM who exhibited discrepancies between forward and reverse blood grouping. Further investigation revealed that the patient's blood type was a variant of blood group B. While type III discrepancies, typically characterized by elevated globulin levels causing false-positive reactions in both forward and reverse blood grouping, are common in multiple myeloma, our case differed due to the loss of B antigens secondary to the malignant condition. This caused a discrepancy in forward blood grouping. The rarity of ABO discrepancies in multiple myeloma underscores the importance of thorough evaluation. Awareness of potential antigen alterations in such patients is crucial to ensure safe transfusion practices.

## Introduction

Plasma cell myeloma (PCM) is a hematologic malignancy characterized by neoplastic plasma cells in the bone marrow that produce monoclonal paraprotein [1]. PCM accounts for 10% of hematopoietic neoplasms and typically affects individuals in their late middle age and older [1]. Studies have indicated that exposure to ionizing radiation may increase the risk of developing PCM. The common presenting features of PCM are associated with end-organ damage, manifesting as one or more of the following: hypercalcemia, renal insufficiency, anemia, and bone lesions, collectively known as CRAB [2,3]. In PCM, ABO discrepancies in blood group tests can occur due to protein abnormalities that cause pseudo-agglutination or rouleaux formation. Here, we report a case with unusual ABO discrepancies in a patient diagnosed with PCM.

## Case presentation

A 51-year-old male presented to the hospital with complaints of lethargy and exertional dyspnea for six months, associated with lower back pain for one month and a weight loss of 20 kg over five months. Laboratory investigations showed a hemoglobin level of 6.8 g/dL, WBC count of 9.65 x 10^9^/L, and platelets of 221 x 10^9^/L. Peripheral smear examination revealed severe anemia with a leucoerythroblastic picture and the presence of rouleaux formation. Serum calcium was elevated at 2.8 mmol/L, blood urea nitrogen at 8.2 mmol/L, and creatinine at 164 umol/L (Table 1). Skull X-rays showed multiple lytic lesions over the skull. The differential diagnosis included plasma cell myeloma or metastases; thus, a bone marrow study was planned. The aspiration yielded a dry tap, but the bone marrow biopsy showed plasma cells replacing the marrow, confirming plasma cell myeloma.

**Table 1:** Other laboratory investigations conducted for the patient during this hospital admission.

Parameters	Result	Normal range (Unit)
Hemoglobin (Hb)	6.8	12.0-15.0 g/dL
White Blood Cell (WBC)	9.65	4-10 x10^9^/L
Platelet	221	150-410x10^9^/L
Calcium	2.8	2.2-2.7 mmol/L
Urea	8.2	2.8 7.2 mmol/L
Creatinine	164	45 - 84 umol/L

**Figure 1:**
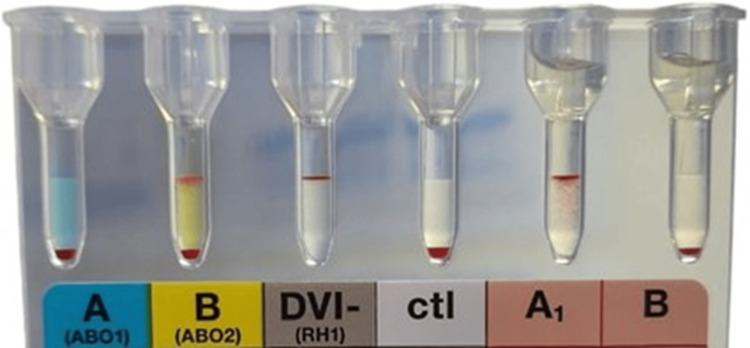
Blood grouping results from the gel card method showing mixed-field agglutination with anti-B.

Due to low hemoglobin levels, blood transfusions were recommended. A blood sample was sent for blood typing and crossmatching. Blood grouping was conducted using the tube method and gel card method (DiaClon, BioRad, Switzerland). In the forward grouping, negative agglutination was seen with anti-A, and a mixed-field reaction was observed with anti-B. Conversely, a strong anti-A reaction was detected at room temperature in the reverse grouping (Figure 1). The reaction most likely represents subgroup B.

Incubation of the sample at 4°C using the tube method to enhance the detection of weak or cold-reacting antibodies and antigens showed no changes. Additional assessment was carried out utilizing the adsorption-elution agglutination assay. The results revealed that the patient's blood group represents a subgroup B, a variant within the ABO system, as the elution displayed a strong reaction with anti-B, confirming the presence of antigen B on the patient's red blood cells. The findings are outlined in Table 2. An indirect antiglobulin test was also performed using a three-cell panel (ID-Diacell BioRad) with antihuman globulin (AHG) using a low ionic strength solution, which showed a negative reaction.

**Table 2:** Reactions observed when patient RBCs were tested with different antisera at various temperatures using the tube method. 0: No agglutination; mf: Mixed field agglutination.

Test	Forward grouping				Reverse grouping		
	Anti A	Anti B	Anti AB	Anti H	A cell	B cell	O cell
Room Temperature	0	2+/mf	2+/mf	Not done	4+	0	0
4^o^C	0	2+/mf	2+/mf	Not done	4+	0	0
Adsorption elution	-	3+	-	4+	-	-	-
Secretor status	Not done						

## Discussion

ABO blood grouping is pivotal in transfusion medicine. Adhering to the gold standard technique, forward and reverse typing allows for cross-verification of results. Discrepancies arise when the red blood cell (forward) grouping contradicts the serum (reverse) grouping. Such discrepancies may stem from various factors, including clerical or technical errors, as well as pathological conditions that influence blood group antigens or antibodies [4].

ABO discrepancies fall into four main types. Type I discrepancies occur when there are issues in reverse grouping because antibodies are weak or absent. Type II discrepancies occur in forward grouping when antigens are weak or missing. Type III discrepancies are caused by excess plasma proteins. Type IV discrepancies stem from various causes, such as cold autoantibodies, cold alloantibodies, and polyagglutination [1,5].

In our case, we encountered a patient with PCM exhibiting Type II discrepancies. We observed a weak/mixed field reaction in forward grouping but a strong reaction in reverse grouping. Type III discrepancies are commonly associated with PCM, characterized by elevated M protein levels from myeloma cells, which can lead to rouleaux formation and false-positive reactions in reverse grouping. However, other reported cases have highlighted Type I discrepancies, attributed to severe deficiencies of IgM, particularly in blood groups A or B, resulting in weak agglutination reactions. Our case represents a unique instance as the first reported occurrence of myeloma with a Type II discrepancy directly linked to the underlying malignant condition.

Loss or reduced expression of ABH antigens is uncommon and may occur secondary to malignancy. Both solid tumors and hematological malignancies have been linked to changes in A, B, and H antigen expression, affecting forward blood grouping discrepancies [6]. This was initially reported by Logjam et al., who observed reduced ABO antigen expression in a patient previously with normal levels, which was attributed to malignancy [4,7].

Diminished expression of ABH antigens can occur due to variant ABO alleles and hematological malignancies such as leukemia, myelodysplastic syndrome, myeloproliferative disorders, and sometimes Hodgkin’s lymphoma [7]. Myeloid neoplasms are more commonly associated with ABH antigenic alterations than lymphoid malignancies [7]. However, in our case, loss of expression of the B antigens occurs in lymphoid malignancies. Additionally, loss of ABH antigens is also seen in the tumor cells of many types of carcinoma, including bladder, lung, head and neck, cervical, and thyroid [6].

**Figure 2:**
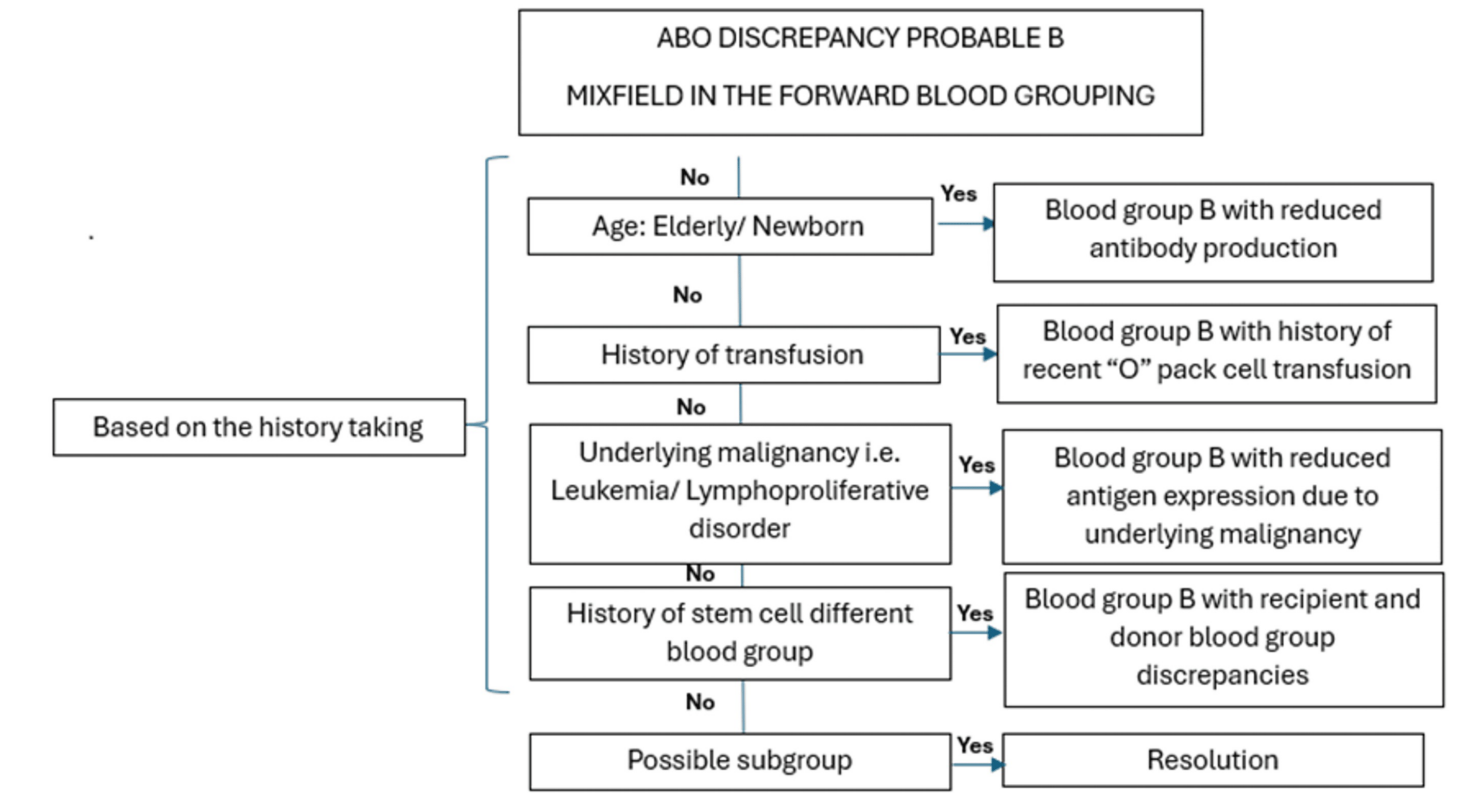
Illustrates the methodology for addressing ABO discrepancies, with particular emphasis on detecting mixed field, weak, or absent antigens during forward grouping. This figure was created by the author.

Subgroup B is uncommon in the general population and is often mistaken for group O because of the minimal presence of the B antigen on the RBC surface. These weak B subgroups, including Bm, B3, Bx, and Bel, are notably rare and require advanced techniques such as adsorption and elution for accurate detection [8,9]. Molecular testing typically follows to confirm and precisely type the subgroups [10]. Resolving blood grouping discrepancies is paramount prior to transfusion or transplantation to prevent potentially fatal reactions. Figure 2 illustrates the methodology for addressing ABO discrepancies, with a particular emphasis on detecting mixed field/weak or absent antigens during forward grouping. This highlights the importance of conducting a comprehensive patient history before concluding that the individual belongs to subgroup B. In our case, after confirming the patient's blood type as subgroup B, we opted to provide group O blood to avoid potential transfusion reactions, as group O is universally compatible and reduces the risk of alloimmunization.

## Conclusions

This case highlights the significance of considering malignancy-associated antigen changes when interpreting ABO blood grouping, emphasizing the necessity for accurate typing methodologies and comprehensive transfusion management strategies in patients with PCM. When such individuals require urgent transfusion and the ABO discrepancy has not yet been resolved, they should receive blood that is crossmatch-compatible with the O blood group.
